# The nonlocal universe

**DOI:** 10.1080/19420889.2020.1822583

**Published:** 2020-10-11

**Authors:** Andrew Lohrey, Bruce Boreham

**Affiliations:** a University of Technology, Sydney (1992); Professional Affiliate, The Galileo Commission, London; b Professor of Physics CQU (1989-98), Fellow of the Australian Institute of Physics

**Keywords:** Local realism, nonlocal realism, locality, nonlocality, meaning, consciousness, paradigm, future of science

## Abstract

We propose that the universe is nonlocal and that the appropriate worldview or paradigm for this understanding is nonlocal realism. Currently the worldview of local realism guides and frames the understanding and interpretations of science. Local realism was the worldview employed by Einstein in his relativity theories, but the principles of this paradigm have operated as the guiding framework for the rest of classic science for more than a century. This paper points to incoherencies in local realism and to the violation of its principles by recent experiments; it suggests that these negative effects have undermined the credibility and legitimacy of this worldview. We offer a more inclusive worldview for the future of science called nonlocal realism. Unlike local realism, the worldview of nonlocal realism encompasses meaning, mind and universal consciousness.

## Introduction

A paradigm or worldview is not a theory, or a series of theories, or a hypothesis. Thomas Kuhn, with whom the now customary use of the term originated, wrote that ‘On the one hand, it stands for the entire constellation of beliefs, values, techniques, and so on shared by the members of a given community. On the other hand, it denotes one sort of element in that constellation, the concrete puzzle-solutions.’ [1] Kuhn also suggested that a paradigm was a ‘disciplinary matrix’ but never satisfactorily resolved the distinction between a constellation and one element of that constellation.

We are applying the notion of a disciplinary matrix to the term ‘paradigm’. Kuhn’s disciplinary matrix represents a network of norms, values and rules common to the practitioners of a particular discipline. A similar view is held by Iain McGilchrist who, in the Foreword to the *Galileo Commission Report*, compares a paradigm with a ‘lens through which we apprehend reality, the problem being that, while such paradigms are indispensable, we tend to be oblivious to the inevitably distorting effect of the lens’.^1^

The idea of a ‘lens’ implies a worldview or paradigm that is not innocent but contains a set of principles, habits of mind and predispositions. Like all other disciplines, scientific practices, whether theoretical, mathematical or experimental, are always subject to a background framework involving hidden assumptions, predispositions and at times a set of principles. A paradigm or worldview operates like a pilot that guides the scientist’s actions and dictates what is observed and measured, how results are interpreted, what research is carried out, what are the appropriate controls and equipment for experiments and finally what and how theories are developed.

These framing conditions are addressed directly in this paper. They are not concerned with scientific methods, experiments or their results, but have a focus on the psychological realities of paradigms. How a paradigm determines certain kinds of interpretations is instructive in how scientific results are understood. How science is interpreted is a subject area outside the conventions of physics, biology, mathematics or medicine, yet it is one that deeply influences every aspect of science.

The psychological reality of hidden assumptions and predispositions that create a paradigm will determine how measurements are conducted, or even what is meant by measurement. The practices of measurement are of central significance for all of science, and so these determining factors are critical to every aspect of science. The hidden assumptions and predispositions of the paradigm of local realism (discussed below] mandates that scientific measurements are essentially physical and ‘good’ measurements should produce factual information that is reliable. What is missing from this paradigm is any consideration of the psychological reality concerning the hidden assumptions and predispositions that have produced this paradigm. These are not inconsiderable factors.

The paradigm that takes account of the psychological reality of hidden assumptions and predispositions we call nonlocal realism. From the viewpoint of this paradigm scientific measurements are products of the mind, and hence all paradigms are essentially psychological. This paper is written from the worldview of nonlocal realism and as a consequence proposes that a paradigm operates as a psychological navigation aid that guides the scientist’s actions and dictates how things are measured and interpreted. From the point of view of nonlocal realism, scientific measurements always have at least two levels: the first is the psychology of the paradigm itself; the second will involve a series of instruments and controls, and that will include the symbols of mathematics, and all of these will be arranged and used according to the sightlines and principles of the paradigm.

## Local realism

The paradigm called local realism represents the dominant lens or worldview through which mainstream mechanical science (and that includes Einstein’s theories of relativity) view the world. This framework of mind has grown out of the culture of scientific activity and philosophical debate over the last three centuries. We argue in this paper that one of the major drawbacks of the paradigm of local realism is it presents a divided and partial picture of a universe that is actually whole and nonlocal. To a large extent this criticism embraces those criticisms within biology leveled at reductionist methods and related to dissecting biological systems into their constituent parts and then expecting this reductive approach to explain the complexity of living systems that have emergent properties.

This expectation ignores the observer’s role in detecting complexity, where the system’s collective behavior gives rise to structural modifications and new hierarchical arrangements [2]. Similarly, in physics, emphasizing the constituent parts at the expense of the whole physical system can lead to oversimplified models and experiments that cannot be extrapolated to accurately describe complex physical systems [3,4]. Emerging systems in physics are examples of complex systems where new and complicated properties emerge in response to collective properties and not to properties of the individual parts [5–7].

However, rather than focusing on the limitations of reductionism in biology or physics we are attempting a broader view that has a focus on the psychology of paradigms. This approach enables us to highlight the overall limitations and partiality of the paradigm of local realism when it is employed by anyone in any discipline, as well as how to identify how these constraints can be overcome. In order to achieve that aim we need to begin by identifying the two basic principles of local realism: the ‘local’ and the ‘real’.

The principle of the ‘real’ in local realism is created by denying the role of the mind while over-valuing the physical world, which is taken to be a ‘reality’ separate from the subjective minds of observers. In terms of the ‘local’, physical object**s** are assumed to be influenced only by their immediate (local) physical surroundings. In physics, for example, a particle is assumed to have properties that are localized in that particle and which cannot influence what happens elsewhere. In biology it is assumed that because living systems are made of atoms this should allow us to explain individual component parts solely in terms of their physicochemical properties. Hence, the paradigm of local realism assumes that individual inanimate physical parts are the primary building blocks of living systems as well as the components of a dead physical universe.

The benefits of the paradigm of local realism are its pragmatic techniques that focus behavior and thinking in ways that provide solutions to practical and technical problems. These are the useful skills and techniques that belong to the technician. The major deficiency of local realism comes from the elevation of these entirely local methods into the general field of philosophy so as to advocate a binary and hence divided worldview, which then becomes applicable to all scientific investigation, physical and social, as well as applying throughout the entire universe. Thus, inherent in these principles of the ‘local’ and the ‘real’ is an absolute dualism that divides and separates the physical world from the minds of humans and importantly, the meanings they make [8, The assumption that mind and body are distinct: 9].

The elevation of local, technical and separating practices into a worldview carries with it the denial of any larger picture involving mind, meaning and the all-embracing context of universal consciousness. In addition, the implications of this worldview reinforce each other in ways that are often incoherent. For example, the absolute dualism of local realism divides the universe into mind and matter. This amounts to the denial that scientists have minds that are embedded within the theories, practices and interpretations they use. Here is the incoherence of the objective approach where it is assumed that the practical scientists have a ‘no-mind mind’.

Along with the absolute dualism of local realism comes a series of binary values and preferences, such as quantity in preference to quality; the physical in preference to the mental; objectivity in preference to subjectivity; explicit details in preference to hidden implicit contexts; controlled variables in preference to hidden and uncontrolled variables; the local in preference to the nonlocal. Such binary thinking tends to become habitual so that interconnected and holistic systems will be treated as if they have gaps, divisions and separations. In addition, local realism has a series of important contextual omissions and deletions which are commonly reflected in scientific practices and investigation. These are the deletions or erasures of meaning, mind and consciousness. When these important contexts are deleted from practices and theory the general result again is the separation of parts within wholes along with the fragmentation of knowledge.

When David Bohm wrote in *Wholeness and the Implicate Orde*r [10] about fragmentation and wholeness he was taking a critical stance against the worldview of mechanical science that focuses on fragments but treats these details as fundamentals. As the term implies, a fragment is part of a whole, but when it is seen as a fundamental feature of the world we change it from being a fragment into a separate and over-valued detail or difference that exists without reference to the overarching contexts of life or consciousness. When these contexts of life and consciousness are ignored or considered non-existent, any part, fragment, difference or feature within a scientific investigation will automatically become over-valued. As Bohm suggests, this kind of thinking is incoherent, while the general denial of context produces the fragmentation of meaning and, as a consequence, of knowledge.

Bohm went on to write that fragmentation is a confusion around the question of difference and sameness and ‘*To be confused about what is different and what is not, is to be confused about everything*’ [10, p. 16]. This is a major category of confusion which we suggest is the hallmark of the paradigm of local realism. This raises the question of the credibility, legitimacy and continuing usefulness of the worldview of local realism as the central psychological guide for mainstream science. These are serious questions about the future effectiveness of this paradigm. Yet such questions do not seem to have had much influence on many scientists. For example, Einstein’s commitment to this scientific paradigm was manifest in his insistence on the entirely local nature of particle connections and his rejection of their nonlocal exchanges, or what he called ‘spooky action at a distance’ [11]. Einstein rejected the idea of ‘instantaneous communication’ between particles because in local reality his theory of relativity forbids any signal to travel faster than the speed of light [12].

## Nonlocal realism

We consider that the future of science rests on changing its psychological reference frame from local realism to that of nonlocal realism. Nonlocal realism is the term we use to represent a holistic and integrated worldview that displays the reality (hence realism) of a singular integrated, interconnected universal field of relationships. The reality of this paradigm is established by the evidence that the universe we perceive and measure is nonlocal and by an analysis of the integrated nature of the meanings we make.

Nonlocal realism is closely related to the view espoused by Erwin Schrödinger that the overall number of minds within the universe is just one.^2^ He went further by suggesting that mind has erected the physical outside world out of its own mental stuff [13]. The interconnected universal consciousness implied by the concept of one mind constitutes the nonlocal, singular implicit reality of a universal consciousness that has embedded within itself the local and explicit conscious mind of each individual.

The paradigm of nonlocal realism denies the absolute dualism inherent within local realism along with the specific separation of mind and the more general psychological tendency to divide and separate all aspects of the world. As a consequence of this emphasis, the primary status that has traditionally been given to an independent physical world disappears. Yet the disappearance of this primary status does not make the physical world disappear. The withdrawal of this status comes from its reconstitution as the secondary effects produced by local minds through the processes of perception and conception. As Lohrey argues, this reallocation of status comes about because the context of universal consciousness is taken into account as the fundamental context; a context in which local minds with perceptions and conceptions of a physical world have a secondary status [14].

The reallocation of the physical world’s status comes about because of the asymmetrical structure of universal consciousness, which stands as the permanent background context on which all of science is writ. Universal consciousness has a structural order in which hidden implicit meaning always has primacy over the secondary status of the explicit meanings of distinctions and differences, and these always involve objects and forms. This order tells us that fragments, parts, objects and forms always arise from wholes (not the reverse), and also that explicit meaning always arises from implicit meaning, and that humans always arise from Nature. This asymmetrical order informs us that consciousness has a local mind and a physical body, rather than the reverse that says the body has a local mind that is consciousness.

However, while individual human minds have a secondary status within the paradigm of nonlocal realism, they are always the ground on which all interpretations and observations are developed. Unlike local realism which ignores or sidelines the human mind or treats it as an entity, nonlocal realism has at its core the human mind as the context for all meaning-making and psychological activity. This means that the observer is never simply a local subjectivity but represents the local entrance into a broader implicit context of nonlocal, implicit universal consciousness.

The status of the observer as context becomes more obvious when we begin to take seriously the evidence that *the only way that we are able to know the physical world (and that includes the brains that neuroscientists study) is by observation and conceptualization, and both of these psychological processes operate within the mind of the observer*. This crucial evidence tends to be dismissed or devalued by local realists.

Hence, any scientific interpretation that is framed by the paradigm of nonlocal realism will take on a different character from those seen through the lens of local realism. First, that difference will be in replacing gaps, divisions and separations with an integrated sense of inclusion and interiority. For example, all relations, both local and nonlocal, are internal to the singularity of universal consciousness and operate entirely within this domain, with no relations existing or functioning outside it. This universal domain of consciousness is not the same as the domain of subjectivity, a term applicable to the dualism of local realism. Thus the subject does not stand as an entity independent of the physical world but, rather, functions as the primary always open local doorway into the implicit knowing of universal consciousness.

We can say then that the paradigm of nonlocal realism automatically has at its center the mind of the observer. To put it somewhat differently, in terms of nonlocal realism the observer becomes the center of the universe and that holds for every observer. How is it possible for the center of the universe to be in different locations? It is possible because each mind has a common foundation of implicit meaning embedded within universal consciousness. This implicit field functions prior to any sense of difference (non-symmetry), and therefore the observer, while always a secondary feature to universal consciousness, will at the same time be the central locale for all potential and actual social exchanges. *One of the important social consequences that flow from this is that all observers are equal in being at the center of the universe, with none left out.*

### What evidence is there that the universe is nonlocal and how does this evidence relate to the creation of a paradigm of nonlocal realism?

To answer that question, we turn first to the mind of the observer, which is where we find the primary evidence for the paradigm of nonlocal realism. The mind of the observer has a similar structure to Bohm’s two orders of the universe: the implicate and explicate orders. We relate these two orders to the structure and function of meaning. Why meaning? Bohm considered that meaning was the essential nature of consciousness [15]. We fully agree, but go further by proposing that meaning is the content of language, mind and consciousness. In this paper we have interpreted Bohm’s two great orders not as physical orders but as orders of meaning and hence as the metaphysical orders of universal consciousness.

As a consequence, Bohm’s implicate order represents a universe-wide, permanently hidden context that is actually full of the implicit relations of meaning, that is, a plenum of implicitness or implicit knowing [14]. Likewise, the explicate order represents that multiplicity of secondary and derivative explicit relations that are located within the conscious and explicit minds of individuals. For mainstream science this explicit order is elevated to ‘the physical world’ and then its differential forms are over-valued and made to appear separate and divided through the deletion of the natural integrating forces of the implicate order. The distinctions between the implicate order (implicit meaning) and the explicate order (explicit meaning) are concerned not so much with the differences between the visible and the invisible, but more generally with the differences associated with two kinds of meaning, knowing and intelligibility.

The two kinds of knowing are discovered within the relationship of Bohm’s two orders and, more directly, with the identical relationship between implicit and explicit meaning. As with Bohm’s two orders, meaning has two poles of attraction that are entirely integrated. These are, i) the pole of implicit meaning, and ii) the pole of explicit meaning. The knowing of explicit meaning comes in the creation of distinctions, differences, contrasts and marks. In contrast, implicit knowing produces links, connections, unities and wholes and represents the hidden content of every context in every area of endeavor. Implicit knowing has the structure and function of symmetry. Explicit knowing is structured by the relations of non-symmetry and asymmetry, which gives rise to distinctions and differences within systems [14]. An example of the symmetry of implicit knowing is the intelligence shown by twin particles who know instantly the orientation of the other.

The poles of implicit and explicit meaning have the further attributes of nonlocality and locality. While both these terms have come from scientific developments, they are not exclusive to science as they represent general terms applicable to all human endeavors, while adding significantly to the scope of our understanding of the surface differences and the depth dimensions of universal consciousness and its relationship to local minds. The pole of explicit knowing always has locality in that it creates a focus on locally situated space and time objects, forms, movements and actions (Bohm’s explicate order). This means that explicit meaning is evident in every conscious action across a wide range of behavior, from mathematics to rock climbing, from child-minding to scientific experimentation. On the other hand, the pole of implicit meaning has a nonlocal, infinite and boundless character of knowing and hence functions as a dynamic movement of hidden and entangled relations across and beyond the distinctions of time, space, and the material body and the physical world.

Like Bohm’s two universal orders, these two kinds of knowing are not binary and do not form the kind of absolute dualism common to local realism; rather they are entirely integrated yet allow for two kinds of emphasis: a local and a nonlocal focus. For example, a nonlocal emphasis produces the view of the universe as an infinite singularity, a single system of nonlocal consciousness that has two major features: a background of permanently hidden, implicit relations (that cannot be measured) and a foreground of revealed explicit differences and forms (that produce measurements). This is the holistic gestalt-like structure of universal consciousness. Within this singular system of universal consciousness every relationship is related to every other relationship. This universal singularity of meaning is reflected in physics by the state that has been called superdeterminism [16].

These two kinds of knowing present us with the choice of where we place the emphasis, whether locally or nonlocally. For local realists that emphasis is always on the local, while for nonlocal realists the emphasis is on the nonlocal. However, quite apart from our choices the structure of this infinitely interconnected singular system of universal consciousness is gestalt-like and remains unaffected by whatever emphasis we employ. What is ‘a gestalt-like structure’? The relationship between Bohm’s implicate and explicate orders and the relationship between implicit and explicit meaning are identical in that in both cases these two features cannot be separated from each other and cannot be treated in isolation. This indicates that both orders and meanings are entirely integrated into a single gestalt-like holism in which every local form will always have hidden implicit features or what in quantum physics has sometimes been called hidden variables.

The word ‘hidden’ can be used in two very different ways. One use comes with the assumption that this is a temporary situation and that what is hidden can be, or may be, revealed later. The other use implies that hidden is a permanent condition and this means there is no possibility under any circumstance of measuring this state. It is this second use of hidden that refers to the character of nonlocal connections, for these are relationships that will remain forever hidden, or, more accurately, remain forever non-explicit. Roughly speaking this is the situation scientists find in relation to the question about a particle’s position or momentum, which cannot be simultaneously measured.

Hence, every explicit particle and every life form will have some permanently hidden implicitness (variables) that represent the background context of universal consciousness, a context in which every particle and every life form is immersed. From a nonlocal worldview these two kinds of knowing mean that fragments (in whatever form they take) are always treated as parts of a larger whole and that every local particle, form or object always operates within a nonlocal system of relationships. A fragment may be a virus, a particle, a mathematical equation, a word, a narrative, money, a house, a city, or the measurements of time or the three dimensions of space. In contrast, the local emphasis of local realism is to treat such fragments, not as fragments but as distinctions without having a qualifying context.

### What is the scientific evidence that supports the paradigm of nonlocal realism?

In the last almost seventy years the scientific evidence for nonlocal connections between entangled particles has directly challenged the credibility of Einstein’s common-sense local realist worldview that prohibits instantaneous nonlocal connections. In 1951 Bohm proposed an experiment using correlated pairs of particles that could test instantaneous communication between them [17,18]. He proposed the creation of correlated states between properties of quantum particles that have a discrete set of possible values (e.g. vertically and horizontally polarized light or light spinning up and down). However, this measurement is not enough to confirm action at a distance. It is still possible that correlation observed between the particles could be due to hidden variables (that is, some property of the particle system that predetermines the outcome of the measurement that is currently hidden from view but may be revealed at a later time).

In the early 1960s John Stewart Bell proposed his now famous theorem that local hidden variables cannot account for all the predictions of quantum mechanics (QM) and that local determinism is incapable of describing what is happening in quantum experiments [19–21]. He developed what has come to be known as ‘Bell’s Inequalities’. These inequalities can be used to determine the difference between how strongly entangled particles would be correlated in the different cases of quantum mechanics alone and entanglement involving hidden variables. Such experiments have been carried out many times over several decades, confirming the QM predictions and consistently demonstrating experimentally that the principles of local realism are violated by nonlocal connections that link entangled particles across vast distances [22–28]. Recent research conducted in 2016 by a worldwide research team led by the Institute of Photonic Sciences (ICFO) in Barcelona has again confirmed these findings.^3^ These results imply that quantum mechanics violates either locality or realism.

In addition to these experiments, the Free Will Theorem of Conway and Kochen [29–31] claims to show that ‘in principle’ correlations between particles cannot be due to hidden variables. Based on a number of axioms, they provide a proof that if experimenters have free will then so must elementary particles. Therefore, the hidden variable hypothesis should be abandoned. This claim has attracted both support and criticism, with some critics suggesting that the theorem applies but in a more restricted sense than its authors propose [32,33]. Further, although the starting axioms have been modified by Conway and Kochen in response to criticism, they still draw on concepts from local reality in limiting the speed of propagation of communication and cannot be assumed therefore to support the concepts of nonlocal realism.

A nonlocal universe would be an interconnected, holistic universe and if this is the basic structure of a nonlocal reality then its implications for science and society are profound and revolutionary. For example, it will mean that contexts are to be considered when doing science, and principally that means the contexts of meaning, language, mind and consciousness. A science in which consciousness was not alien to QM experiments is one in which scientists will not be surprised that one of a pair of photons originating from the same source knows instantly the state of its twin even though they are many kilometers apart. If photons ‘know’ something it indicates they have consciousness and that means the universe as a whole has consciousness and is alive. This is not to say that photons have free will for such an idea fails to take account of the gestalt-like structure of universal consciousness. Free will is an issue about differential and explicit decisions made within the local human mind and it is not an issue related to universal consciousness, which is an infinite field that is entirely implicit [14]. The significance of individual scientists and the particles they experiment with not functioning independently is considered further below in the discussion of Wheeler’s delayed choice experiment.

The possibility of violation of realism has led some scientists to revisit the notion of the world as a simulation or local illusion. For instance, Quantum Bayesianism suggests that there is no objective reality but only a subjective estimation of it [34]. Muller takes this further by suggesting that physical reality is fundamentally observer-relative.^4^ The possibility that the world is a simulation has been pursued by Bostrom, suggesting that we are more likely than not to be living in a computer simulation [35]. More recently Erwin et al. propose that the universe is a self-simulation that might exist as a broad class of possible theoretical models of reality obeying the *principle of efficient language* axion [36]. However, a broad consensus has emerged that it is locality that is violated, not realism, and that QM involves some type of nonlocality, although what is the exact nature of the nonlocality remains controversial [37].

Even though experiments in QM have consistently violated the principles of local realism and upheld the principle of nonlocality and, as we have already argued, the significant omissions of local realism produce only a partial view of the universe, in the face of all this overwhelming evidence there is still a tenacious adherence to these principles by most scientists. We can only assume that the very idea that science is host to the guiding principles of a partial paradigm is so alien to many that they treat any criticism of local realism as a mere detail. As a consequence, many seem unable to tell the difference between a fatal blow to their worldview and what maybe thought of as an incidental embarrassing mistake by Einstein and others.

In contrast to this mainstream view there has been over the last century a wide range of scientists who have given voice to, and provided evidence of, a more inclusive, integrated and holistic universe. An appreciation of the point can be gained from David Bohm’s model of the two great orders of the universe, which provide an integrated structure of a holistic universe.

More recently, in her paper ‘Non-locality as a Fundamental Principle of Reality’, Elizabeth Rauscher sets out to confirm the proposition presented in the title of her paper and for the most part succeeds [38]. Yet we are left to wonder what is the ‘reality’ to which she refers. Rauscher does not address this question directly but seems to imply that the reality she refers to is an interconnected, holistic universe, for she writes, ‘It is clear that this principle of nonlocality has profound implications about the nature of a nonlocal universe’. *She goes on to suggest that locality maybe considered as a ‘special and limiting case’ of the more basic nonlocality*.

Some early pioneers of quantum mechanics [39–41], including the mathematician John von Neumann, advanced theories and postulates concerning the possible role of consciousness in the collapse of the wave function and the measurement problem while arguing that the human mind has a direct influence on the collapse (the von Neumann–Wigner interpretation). Today these ideas remain contentious and the subject of debate [42,43]. Wigner originally argued that experimenter consciousness was essential for wave function collapse but moved away from this interpretation in later life [44].^5^ While the term ‘know’ implies consciousness, the well-established nonlocal connection between twin particles suggests a holistic universe that is interconnected through consciousness. Yet a universe that has a background of instant interconnecting consciousness is not the type of nonlocal universe that Rauscher argues for, as she believes that ‘superluminal signals must be invoked’ even though she refers to the universe as ‘nonlocal’.

Rauscher’s proposed nonlocal universe appears to be interconnected by superluminal (faster than light) signals rather than implicit knowing. However, it is commonly agreed that in quantum phenomena, superluminal signaling is impossible in practice. Moreover, many scientists believe that the so-called ‘no-communication theorem’ or ‘no-signalling principle’ excludes superluminal signaling in principle as well as in practice. There are a number of proofs of this theorem to be found in the literature [37, p. 25; 45],^6^ for example, proposing superluminal signaling invokes locality (signals are local and potentially measurable] while on the other hand contradicting locality with the concept of superluminal exchanges. In addition, if nonlocality is a fundamental principle of the universe what is it about faster-than-light signals that is nonlocal? Surely, a faster-than-light movement can be measured to make it so, which in turn makes it entirely local. These are interesting issues because they again raise the question of the psychological framework we are using to understand the nature of nonlocality.

The idea of quantum nonlocality was introduced by Niels Bohr in 1935. To allow *the possibility* that when two particles interacted it might be possible, by measuring one particle, to work out some of the properties of the other without needing to measure it directly (that is, through their entangled history), he suggested that the state of both particles simply became ‘real’ at the same time, that is to say, measuring one particle would instantaneously influence the other, regardless of the distance separating them [46]. This distance can be extremely large, even thousands of light years.

This proposition implied more than just a set of correlations between distant events for it includes the notion that it becomes impossible to measure the features of one particle (position, momentum, polarity) without instantly steering the other into a corresponding state even across vast distances. This means that correlated particles are no longer separate particles, one influencing the other, but two parts of a single system separated but interdependent in space [47]. (or in other words entangled particles are an example of an emergent system).

We see from Bohr’s description that entanglement of a state shared by two particles is a necessary but not sufficient condition of quantum nonlocality. There exist some states that are genuinely entangled but admit a local model [48]. Entanglement is more commonly viewed as an algebraic concept, noted for being a *prerequisite* to nonlocality, whereas nonlocality is defined according to experimental statistics. As we have seen, nonlocality allows two distant observers to obtain experimental statistics that cannot be described by any classical common cause, as if they *instantly* swapped details on this experimental intrusion across a distance^7^ rather than propagating continuously in space-time This means that quantum nonlocality is defined in terms of the processes it allows, not by its fundamental nature.

Berkovitz has examined the proposition of nonlocality in detail as well as an alternative common view that these influences are due to some type of holism and/or non-separability of states of composite systems, which are characteristic of systems in entangled states (like the spin singlet state), and which exclude the very possibility of action at a distance [45,49, p. 8]. Holism is essentially the idea that at least some properties of wholes are not determined by the physical properties of their parts. There are various forms of holism in the literature. For example, Berkovitz defines holism as a violation of the condition of Particularism, that is, **the proposition that** the world is composed of individuals. All individuals have non-relational properties and all relations supervene upon the non-relational properties of the relata and the spatiotemporal relations between them.

Berkovitz considered in detail the nature of holism and non-separability as manifested by various interpretations of quantum mechanics and whether these interpretations predicate the existence of action at a distance. He concludes that quantum action at a distance would be explained by the holistic nature of the quantum realm and/or non-separability of the states of the systems involved where action at a distance is defined as ‘a phenomenon in which a change in intrinsic properties induces a change in the intrinsic properties of a distinct system without their being a process that that carries this influence contiguously in space and time’. Conditions are identified under which all three of holism, non-separability and action at a distance apply and under which action at a distance only is involved. According to Berkovitz all the collapse models postulate the same kinds of holism and non-separability as orthodox quantum mechanics.

In summary, the interpretations of QM considered by Berkovitz involve characterizations of holism, non-separability and/or some type of action at a distance. While holism, non-separability and action at a distance could explain the process of *nonlocality obtained empirically*, they do not address its basic nature or cause, that is, the nature of its connections.

While the physics of nonlocality have been repeatedly verified over the last century, its oblique definition has remained a problem to most scientists. Perhaps this is because one of the defining characteristics of nonlocality appears to be its unmeasurable nature, that is, nonlocality is impossible to measure in any way (see for example Berkovitz and Hemmo^8^). If something is unmeasurable then mainstream science, which rests on the worldview of local realism, is unable to deal with this situation because the measurement of quantities is the raison d’être for this brand of science. Hence, if two particles know instantly the orientation of the other, even though they are many kilometers apart, the instantaneous knowing represents the area that is unmeasurable.

An instantaneous knowing (even by particles) is unmeasurable because in this domain of knowing there are no distinctions or differences that can be measured. In other words, we suggest that any measurement of any kind can only be made when there is a field, space or system containing distinctions and differences that may be compared and correlated. Such comparisons and correlations constitute the processes of measurement. Thus, as a general rule we can say that any measurement, scientific or otherwise, cannot occur in the absence of differences or distinction. Instantaneous connections are a case in point. Another more human case is insight, intuition and extrasensory perception, processes that do not involve differences that can be measured, perhaps the reason why these human abilities are comprehensively dismissed by local realist scientists.

If the first characteristic of a nonlocal connection is the impossibility of measurement, then a second characteristic represents the hidden nature of these connections. We have previously noted that the character of nonlocal connections is such that they will remain forever hidden. There are further distinctions to be made regarding the hidden character of nonlocal connections. These involve the conditions of the visible and the invisible. This distinction relates directly to the two major functions of the human mind: perception and conception. Concept formations, that is, thinking, represents the processes of interpretation, yet these processes are invisible to us for they do not register with the naked eye and hence are hidden from direct sight. Images of visual perceptions, however, are by their very nature visible. As a consequence, one of the critical differences between perceptions and conceptions is the distinction between the visible world and our invisible interpretations of that world. This difference is significant as it allows for the wide variety of interpretations of the same images of the world.

In relation to the processes of knowing these are entirely hidden in the sense that they are not visible. Yet while invisible, the processes of knowing (and we can include the term ‘intelligibility’) are the very basis by which we humans know and make meaning of the world. This is also the case for relationships. Relations are known to us because we use them all the time to make decisions based upon comparisons, assessments, measurements and interpretations. These kinds of relations are explicit. Yet even explicit relations tend to be invisible because they do not register visually for us, though they are known to us. We can realize that two particles can know instantly the orientation of the other and yet such knowing is permanently invisible to us and hence these connections will be unmeasurable. These kinds of relationships have the character of nonlocal connections and again will be permanently invisible.

Rauscher refers to ‘a nonlocal universe’ as one in which nonlocality is ‘a’ fundamental principle. Yet for the paradigm of nonlocal realism it is not so much ‘a’ but ‘the’ fundamental principle. As a consequence, we would expect that a nonlocal universe will be the same everywhere and exhibit the character of nonlocality in every place and every aspect of the universe. What further evidence is there that can substantiate this claim?

We find evidence of the observer as the central context in John Wheeler’s delayed choice experiment.^9^ In Young’s classic two slit experiment when electrons are aimed at a barrier containing two slits, the electrons behave like waves, that is, after traversing the two slits, they at once produce an interference pattern on a detector on the far side of the barrier. If, however, the slits are closed off one at a time the interference pattern disappears, and the electrons pass through the barrier like individual particles. In Wheeler’s delayed choice experiment, the experimenter decides to close one slit or leave both open not before but *after* the electron passes through the barrier, with the same results as when the decision is made before the electron passes through the barrier. That is, the electrons seem to know in advance how the experimenter will choose to observe it. Wheeler’s delayed choice experiment has been experimentally realized by Jacques et al.^10^

In terms of local realism, the results of the delayed choice experiment make no sense at all. However, from the perspective of nonlocal realism these results synchronize with the principles and sightlines of this paradigm. This is because universal consciousness is omnipresent, ‘beginning at the level of the most basic quantum particles … and proceeding to produce a deeper understanding of the entire cosmos’ [50, quoted in 14, p 42]. This means that local individual scientists, along with the electrons they are experimenting with, do not function in isolation, rather they both exist within the nonlocal, implicit context of a knowing universal consciousness. If particles know in advance how the experiment chooses to observe them, they know because universal consciousness is immanent within the scientist’s mind as well as in the particles of the experiment.

One of the oldest interpretations of QM is the Copenhagen interpretation devised in the 1920s by Niels Bohr and Werner Heisenberg. Some of the effects of nonlocal realism can be appreciated when we apply it to the Copenhagen interpretation. According to this interpretation, physical systems do not have definite differential and explicit properties until measured by local minds. This is an accurate description of the functions of the human mind. However, local minds do not exist on their own as assumed by local realism, but only exist within the context of nonlocal consciousness, which has a foundation role to play in all local measurements.

In the vocabulary of QM, the act of measurement affects the system, causing a set of probabilities to reduce to one *after* measurement. Measurement is defined in local realist terms as the testing or manipulation of a physical system in order to yield a numerical result. Bohr believed that the results of measurement could only be described in the language of classical physics and that it made no sense to ask what was going on in the invisible quantum realm. From the worldview of nonlocal realism, he is entirely correct; it makes no sense to try and describe distinctions within the invisible unmeasurable realm of universal consciousness because there are no conscious distinctions within this domain. In this domain there are only hidden relations, and the human uncertainty that always attends nonlocal implicit exchanges.

Another window into the dilemma of local reality is obtained from the more minimalist interpretation of quantum determinism given in the statistical interpretation or ensemble interpretation of QM.^11^ This is based on Max Born, The Statistical Interpretation of Quantum Mechanics, Nobel Lecture, December 11, 1 In this interpretation the probability distribution function is taken at face value. This function is used only to predict probabilities. The behavior of an ensemble of particles can be predicted but not the behavior of individual systems or particles.

In order to know the outcome for an individual particle the experimenter must observe it, that is, the role of the observer is passive. There is no physical object or process that collapses because the wave function has no physical meaning. The problem that there is no prescription in QM for the collapse of the wave function is bypassed. While this has resolved one dilemma it introduces another. In this view quantum physics is unable to provide a deterministic outcome and we arrive back at Bohr’s view where we have nothing more to say about quantum reality, or we say that the Copenhagen interpretation is simply a metaphor for the mathematics. In either case it is not possible to know in advance the outcome of a measurement.

We have effectively arrived at a limit of understanding where according to Bohm ‘the statistical features of quantum theory are thus regarded as representing a kind of irreducible lawlessness of *individual* phenomena in the quantum domain. All individual laws (classical physics) are then regarded as limiting cases of the probability laws of quantum theory, approximately valid for systems involving large numbers of molecules.’ [10, p. 69] From the perspective of nonlocal realism, Bohr was correct insofar as the results of measurement could only be described in terms of classical physics.

However, the vocabulary of nonlocal realism answers this difficulty in that the unmeasurable relations of meaning operate prior to the visible macro world. The gestalt-like structure of this metaphysical domain of consciousness allows for both a description of the ‘invisible quantum realm’ as well as an integrated description of the measurement results. In regard to the invisible quantum realm this is the hidden universal realm of implicit knowing called consciousness. In relation to the language of classical physics this is directed at the multiple and differential realm of Bohm’s explicate order involving explicit distinctions, in which the physical behaviors of the experimenting scientist take place.

There are not two independent orders involved in scientific investigations or in life. There is only one. The so-called ‘potential realities’ of the invisible quantum realm represent the implicit meanings and knowing of the implicate order, which is the fundamental nonlocality of the universe. The local realist view concerning the measurements of the physical world do not have the status of an independent order, for such measurements and their discourses represent only a set of derivative distinctions and differences that arise from the prime order of universal consciousness. Measurements are explicit maps that have their semantic foundations within this prime order of knowing.

In addition, there is no physical object or process that collapses with the exchanges of these two orders of meaning, so the phrase ‘the wave function collapse’ appears to be a local realist construction and overly physical and incongruous. What continually occurs between these two orders of meaning are the transformations of implicit meaning into explicit meaning: transformations that contain the structure of *implicit-to-explicit* exchanges and represent the key feature that gives rise to the local conscious mind of the individual. This significant change from nonlocal to local, from implicate to explicate, from implicit to explicit meaning has under the guidance of local realism undergone a rough categorization as ‘the wave function collapse’. These transformations do not occur externally, *out there* in the physical world, but operate entirely within the singularity of universal consciousness while involving the local level of the human scientist’s mind.

For the scientists who employ nonlocal realism to organize their work it will mean that all experiments will take place within a nonlocal as well as a local world. In other words, every experiment in whatever discipline will have classic, macro, local time and space objects, movements and measurements operating concurrently with nonlocal, unmeasurable interconnections of universal consciousness of which the scientist’s mind will be a part. This integrated duality tells us that within every scientific experiment there are not two contradictory conceptual schemes at work (micro and macro) as many local realists have assumed. Rather, nonlocal realism takes account of the encompassing singularity of universal consciousness and that unifying reality involves relations that are implicit and nonlocal as well as those that are explicit, practical and local. In other words, nonlocal realism embraces invisible, unmeasurable relations as well as those visible objects and forms associated with explicit and differential relations.

For the bench scientist this means overcoming the constraints of local realism by placing greater emphasis on the nonlocal, that is, addressing the entire context of an experiment via a holistic and inclusive approach that recognizes connectedness, complexity, the role of the collective properties of emerging systems, and the role of consciousness. It also requires that experimental controls be developed and exercised that are appropriate to the broader more holistic approach of nonlocal realism [51,52].^12^ Nonlocal realism does not provide the scientist with a set of levers that can be pulled to control an experiment better and nor does it give a sharper definition to binary logic. Rather, by employing the psychological navigational aid of nonlocal realism we become more aware of the unities and affinities that connect everything to everything else. For example, [53], argues that space and time are not physical, rather the differentials of space and time are always simply aspects of the local human mind, while the symmetry that underlies both is a central and unmeasurable feature of universal consciousness. Both these local and nonlocal features form the integrated systems we call space and time or the spacetime continuum.

Applied to biology, the nonlocal realist would not refer to ‘life’ when looking through the electron microscope at the structure of a bacterium. The integrative reference here should be a local ‘life form’. The general designation ‘life’ properly applied refers to that animate field of universal consciousness because life is one of its characteristics. Plant neurobiology is a recently developed field of plant biology research ‘aimed at understanding how plants perceive their circumstances and respond to environmental inputs in an integrated fashion’ [54]. This field of research brings up the controversial question of ‘plant intelligence’ and the less controversial newly developed field of bioinformatics which seeks to explain plant communication, now well established as plant signaling.

We would speculate that by applying a nonlocal approach to plant neurobiology research and specifically to the problems of plant communication this change would be more productive than using the orthodox paradigm of local realism. One example of that change would be the deletion the term ‘information’ from research and replacing it with ‘meaning’ [55]. ‘Information’ is a mechanical, local realist term, the separating theory of which tends to prevent deeper understandings of the implications inherent within any and all forms of communication. Hence, when communication is understood to indicate an exchange of meaning the latent unities and integrated connections within communication exchanges between organisms of any kind will tend to become more obvious. These integrative aspects in plant communication should also assist with the controversial question of plant intelligence because the sightlines within such nonlocal observations inherently take account of consciousness and hence would not be inhibited by local realist predispositions for divisions and separations. In other words, the ‘controversy’ about plant intelligence is generated by the worldview of local realism and so it does not come from any scientific evidence.

Thus, scientific investigations and interpretations framed by nonlocal realism will have a very different character to classic local realist ones. Under the influence of nonlocal realism, the universe is no longer a dead, insentient world but rather an animate participatory universe where every single physical object, from particles to galaxies, from microbes to monkeys will contain more hidden connecting meaning than what is revealed by the appearance of their physical shapes, exchanges and movements.

Finally, an example of nonlocal realism comes from the inventor and philosopher Arthur Young (1905–1995), who stated that photons have no mass, charge or time, which suggests their behavior transcends physics in the manner that consciousness does [56]. Young also wrote that ‘Light is not like other things … ’ and ‘Light is not seen; it is a seeing [56, p. 11].’ When light becomes a ‘seeing’ there is no more dualism of subjective seer and objective seen, rather there is simply a process within the singularity of an interconnected universe that jointly involves a local mind along with nonlocal consciousness. What is given in this participatory process is the sight within seeing, that is, the seeing essence of visual perception. Yet the ownership of that sight does not come from a separate local mind or the intention of an individual who has almost no control over seeing or not seeing. Rather, the ownership and agency of the sight within our seeing entirely rests with nonlocal universal consciousness.

## Conclusion

Our review of the paradigms of local realism and nonlocal realism suggests that the usefulness of local realism as a worldview is severely limited and that science in the future will most likely turn to a more inclusive, integrated paradigm that has the character of nonlocal realism.

## Notes

[1] https://explore.scimednet.org/

[2] https://www.organism.earth/library/quotes/178.

[3] https://thebigbelltest.org/science/(The Big Bell test of 2016); Handsteiner J [57]).

[4] Muller 2019: arXiv:1712.01826

[5] John Wheeler also believed that consciousness plays a role in bringing the universe into existence – the Participatory Anthropic Principle (PAP) (‘The anthropic universe’ Abc.net.au. 2006–02-18. Retrieved 2011–01-24). In contrast, Bohr rejected the idea that the experimental outcome is due to the observer (Faye J [58] ‘It is certainly not possible for the observer to influence the events which appear under the conditions he has arranged’ (Bohr N [59]). David Bohm himself developed a theory of QM (the pilot wave theory or the de-Broglie-Bohm theory). This theory is nonlocal (thereby satisfying Bell’s inequality), taking place in a single space-time and is deterministic. The measurement problem is resolved because particles have definite positions at all times [60].

[6] https://en.wikipedia.org/wiki/No-communication_theorem

[7] https://en.wikipedia.org/wiki/Quantum_entanglement

[8] See Sections 8.1 and 9 in **[**61].

[9] This is discussed in John Horgan’s (2018) article on Wheeler: ‘Do our questions create the world? (https://blogs.scientificamerican.com/cross-check/do-our-questions create the world/); Wheeler JA [62].

[10]The experimental realisation of Wheeler’s delayed-choice Gedanken Experiment. In V [51], *Science*, **315**[5814), pp 966–8. Also, in D [52]. Consciousness and the double-slit interference pattern: Six experiments. *Physics Essays* **25**(2]. They describe using a double-slit optical system to test the possible role of consciousness in the experiment. In this experiment the ratio of the interference pattern’s double-slit spectral power to its single-slit spectral was predicted to decrease when attention was focused toward the double slit as compared to away from it. Data contributed by 137 people in 6 experiments indicate that the spectral ratio decreased as predicted and it was concluded these results were consistent with a consciousness-related interpretation of the quantum measurement problem.

[11]This is based on Max Born, *The Statistical Interpretation of Quantum Mechanics*, Nobel Lecture, December 11, 1954. Bohr accepted Born’s statistical interpretation because he believed that the wave function has only a symbolic meaning and does not represent anything real. Therefore, it makes no sense to talk about the collapse of the wave function (Faye J [58], p. 7). Bohr stressed the importance of experiments to understand the quantum world. Each experiment in effect asks a question of the quantum world. The questions are highly colored by everyday experience and the experiments are rooted in classical physics. (Gribbin J [63]).

[12]https://www.embopress.org/doi/10.15252/embr.201949110
